# Improving Surgical Outcome Using Diffusion Tensor Imaging Techniques in Deep Brain Stimulation

**DOI:** 10.3389/fsurg.2017.00054

**Published:** 2017-09-28

**Authors:** Angela An Qi See, Nicolas Kon Kam King

**Affiliations:** ^1^Department of Neurosurgery, National Neuroscience Institute, Singapore, Singapore; ^2^Duke-NUS Medical School, Singapore, Singapore

**Keywords:** deep brain stimulation, diffusion tensor imaging, diffusion tractography, magnetic resonance imaging, surgical outcomes

## Abstract

**Introduction:**

Recent advances in surgical imaging include the use of diffusion tensor imaging (DTI) in deep brain stimulation (DBS) and provide a detailed view of the white matter tracts and their connections which are not seen with conventional magnetic resonance imaging. Given that the efficacy of DBS depends on the precise and accurate targeting of these circuits, better surgical planning using information obtained from DTI may lead to improved surgical outcome. We aim to review the available literature to evaluate the efficacy of such a strategy.

**Methods:**

A search of PubMed was performed to identify all articles using the search terms “(diffusion tractography OR diffusion tensor imaging OR DTI) AND (deep brain stimulation OR DBS).” Studies were included if DTI was used and clinical outcomes were reported.

**Results:**

We identified 35 studies where the use of DTI in DBS was evaluated. The most studied pathology was movement disorders (17 studies), psychiatric disorders (11 studies), and pain (7 studies). The overall responder rates for tremor reduction was 70.0% (SD = 26.1%) in 69 patients, 36.5% (SD = 19.1%) for obsessive–compulsive disorder in 9 patients, 48.3% (SD = 40.0%) for depression in 40 patients, and 49.7% (SD = 35.1%) for chronic pain in 23 patients.

**Discussion:**

The studies reviewed show that the use of DTI for surgical planning is feasible, provide additional information over conventional targeting methods, and can improve surgical outcome. Patients in whom the DBS electrodes were within the DTI targets experienced better outcomes than those in whom the electrodes were not. Many current studies are limited by their small sample size or retrospective nature. The use of DTI in DBS planning appears underutilized and further studies are warranted given that surgical outcome can be optimized using this non-invasive technique.

## Introduction

Deep brain stimulation (DBS) is an established therapy for the treatment of medically refractory movement disorders including Parkinson’s disease (1–3), essential tremor (4), and dystonia (5, 6). In recent years, it has gained increasing use as a treatment modality for psychiatric disorders such as depression and obsessive–compulsive disorder (OCD) (7–9), obesity (10), and memory disorders (11), as well as pain (12).

Conventional DBS surgical planning has been based on direct or indirect targeting combined with intraoperative electrophysiological recordings in order to locate targets in the deep brain structures. However, in indirect targeting, stereotactic coordinates derived from 2D histology-based human brain atlases are prone to spatial distortions. Further, in direct targeting, many DBS targets are in the internal subdivisions of thalamus and direct targeting or visualization using MRI is not feasible. Standard MRI also does not visualize white matter tracts.

Recent advances in surgical imaging include the use of diffusion tensor imaging (DTI) for surgical planning providing the surgeon with a detailed view of the white matter tracts and their connections. Diffusion tractography refers to 3D models of white matter pathways generated from diffusion weighted data, most commonly diffusion tensor imaging (DTI). Although diffusion tractography potentially suffers from a number of limitations including poor resolution, inaccuracies introduced by poor signal-to-noise ratio, possible misregistration with anatomic images, and inability to resolve complex fiber crossing [see Mori and van Zijl (13) for a technical review], it has been successfully used to model neuronal connections.

There is increasing evidence that diffusion tractography might yield reliable and reproducible results in DBS if it is performed under certain controlled conditions. Previous studies have evaluated the impact of integrating diffusion tractography-based studies in DBS, but these articles were mainly focused on the concept of diffusion tractography, technical considerations of diffusion methodologies or utility of diffusion imaging in target selection and optimization of electrode placement intraoperatively [for reviews, see Calabrese (14) or Torres et al. (15)]. In this study, we aim to review the available literature to evaluate the efficacy of using diffusion tractography in DBS to improve postoperative outcome.

## Methods

### Search Strategy

An electronic search using PubMed up to July 2017 was performed to identify articles for inclusion using the key words “(diffusion tractography OR diffusion tensor imaging OR DTI) AND (deep brain stimulation OR DBS).” In addition, the reference lists of all selected studies were reviewed to further identify potentially relevant studies. Duplicate searches were eliminated. All studies were screened based on their titles and abstracts. Abstracts, conference presentations, editorials, reviews and expert opinions were excluded. Full text of the studies identified in the search process was used to further assess for inclusion.

### Selection Criteria

Studies had to fulfill the following criteria to be included: (i) Involve human participants who have undergone deep brain stimulation, (ii) DTI data were acquired pre- or postoperatively, (iii) DTI data were utilized in DBS planning, or analyzed retrospectively, (iv) postoperative clinical response outcomes for the indicated use of DBS was reported, and (v) articles in the English language. Studies which reported the side effects of DBS without reporting the clinical response for DBS were excluded. Studies which reported the same series of patients were analyzed collectively and their quantitative assessment were consolidated. If different DTI analytic approaches were used on the same group of patients, these assessments were separately analyzed.

**Figure 1:**
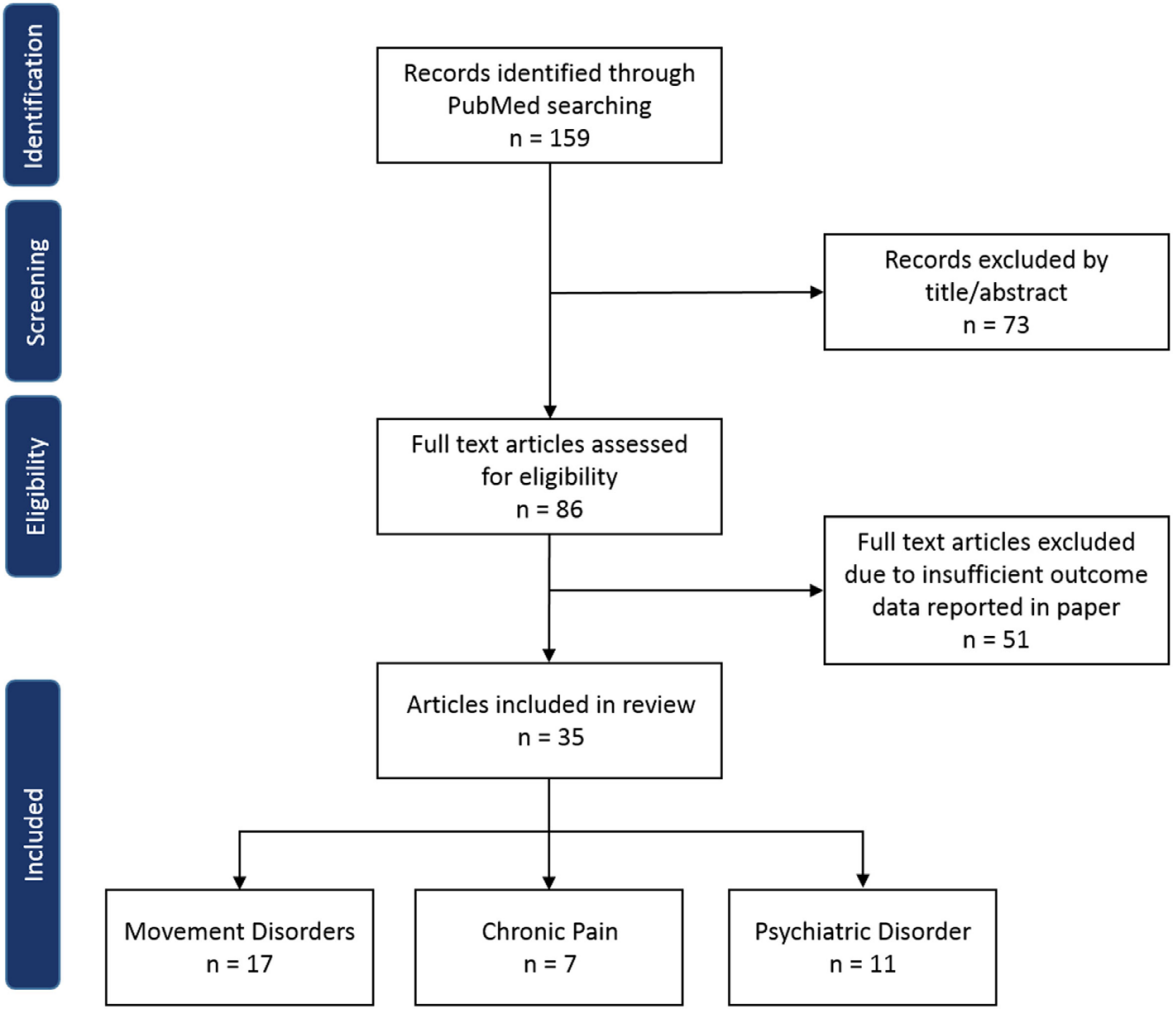
Flow diagram of literature review.

A total of 159 studies were identified through PubMed electronic search and from analysis of reference lists. After exclusion of duplicate or irrelevant references, 86 potentially relevant full text articles were retrieved for detailed evaluation. Of those articles, 35 studies met the inclusion criteria and were included in this review (Figure 1).

The studies were categorized according to their indications for DBS, DTI analysis approach, fiber tractography method, and whether these were retrospective or prospective studies. The indications for DBS included tremor, dystonia and freezing of gait for movement disorders, depression and obsessive–compulsion for psychiatric disorders, as well as various pain disorders. The two types of DTI analytical approaches have been described by Calabrese (14). Briefly, in tract stimulation modeling, the region surrounding an implanted DBS electrode was used as a seed for diffusion tractography to identify the population of brain connections which are likely to be modulated by a given DBS contact. In tract proximity analysis, the location of DBS contact was analyzed with *a priori* knowledge of a tract of interest to investigate whether its proximity with an electrode contact is correlated with treatment efficacy or to evaluate the accuracy of electrode targeting. Both approaches are retrospective in nature, contrary to direct tract targeting where DBS electrodes are targeted prospectively based on diffusion tractography-derived tracts of interest. Similarly, two categories of tractography methods were commonly used and include probabilistic or deterministic fiber tracking. Briefly, in deterministic tracking, the principal direction is followed bidirectionally from a seed point to generate the same set of tracts each time and assumes a dominant fiber orientation in each voxel. In the more computationally expensive and iterative method of probabilistic tracking, the full spherical function is used to estimate the probability distribution of fiber orientations. The probability is defined in a number of ways based on the algorithm used and this probability governs the reconstruction of the fasciculi. The advantages and technical limitations of these methods in DBS have been discussed extensively in existing literature [see Calabrese (14) for a recent review].

The classification of patients into responder or non-responder groups was based on their original classifications in the studies. Responder rate within each indication was calculated across all studies by dividing the total number of responders over the total sample size. Reported improvement in measures appropriate for assessing the specific symptoms indicated for DBS was used to calculate the percentage mean improvement for each study. In studies where more than one measure was reported, the data from the measure demonstrating the greatest improvement were used in the calculation for overall mean improvement. Overall percentage mean improvement within each indication was calculated by averaging the mean improvements across all studies.

## Results

### Overview

**Figure 2:**
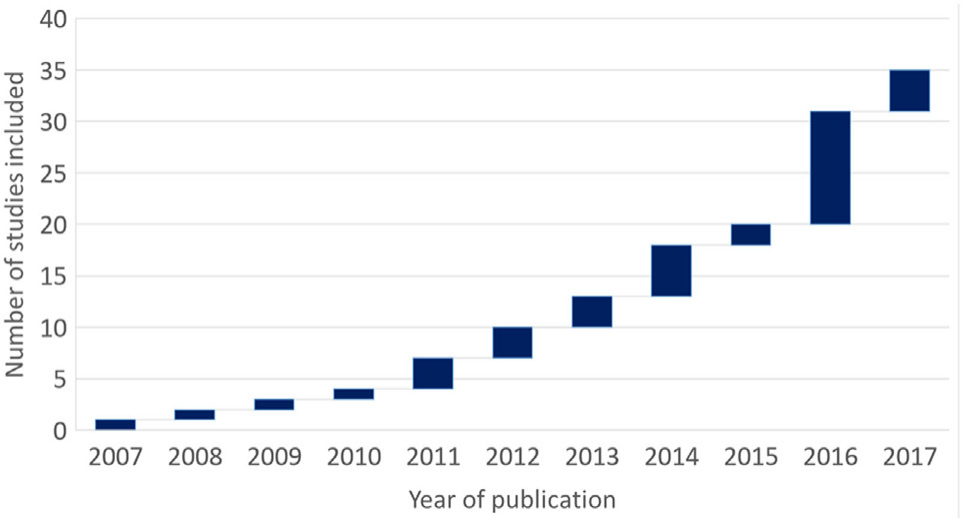
Waterfall diagram of studies included in this review by year of publication over a decade.

Publications involving the use of DTI in DBS have increased steadily over the past 10 years, with a surge in numbers observed in 2016 (Figure 2). The studies included in this review can be found in Table 1. Out of the 35 studies, 17 (49%) were for medically refractory movement disorders, 3 (9%) for OCDs, 8 (23%) for depression, and 7 (20%) for chronic pain.

**Table 1:** List of studies included in this review (*N* = 35).

First author, year (ref)	*N*	Indication	Age (years)^a^	Approach	Method	Follow-up (months)
Fenoy, 2017 (16)	20	Tre (ET)	66.8 ± 10.5	DTT	D	n.r.
Coenen, 2017 (17)	1	Tre (ET)	72	DTT	D	n.r.
O’Holloran, 2016 (18)	2	Tre (PD)	63.0 ± 2.8	DTT	P	24
Coenen, 2016 (19)	2	Tre (PD)	75.5 ± 0.7	DTT	D	5–8
Coenen, 2012 (20)	1	Tre (ET)	73	DTT	D	18
Coenen, 2011a (21)	1	Tre (Dys)	37	DTT	D	3
King, 2016 (22) and Sammartino, 2016 (23)	6^b^	Tre (ET/PD)	71.9 ± 5.9	TPA	P	12
Coenen, 2014 (24)	9^c^	Tre (ET/PD/Dys)	64.2 ± 17.1	TPA	D	3–17
Sweet, 2014 (25)	7^d^	Tre (PD)	62.9 ± 7.3	TPA	D	n.r.
Coenen, 2011b (26)	1	Tre (PD)	73	TPA	D	6
Boccard, 2016a (27)	1	Tre (post HI)	20	TSM	P	6
Klein, 2012 (28)	12	Tre (ET/PD)	64.8 ± 11.0	TSM	P	1–3
Pouratian, 2011 (29)	6	Tre (ET)	69.3 ± 6.1	TSM	P	n.r.
Schweder, 2010 (30)	1	FOG (PD)	56	TSM	P	n.r.
Rozanski, 2014 (31) and Rozanski, 2017 (32)	10	Dys	59.1 ± 13.7	TSM	P	2–4
Coenen, 2016 (33)	2	OCD	41.5 ± 13.5	DTT	D	12
Makris, 2016 (34)	1	OCD	30	TPA	P	6
Hartmann, 2016 (35)	6	OCD	36.2 ± 8.6	TSM	P	24
Fenoy, 2016 (36)	4	Dep	46.3 ± 8.9	DTT	D	4.5
Schlaepfer, 2013 (37)	7	Dep	42.6 ± 9.8	DTT	P	3
Tsolaki, 2017 (38)	2	Dep	n.r.	TPA	P	n.r.
McNab, 2009 (39)	1	Dep	60	TSM	D	12
Accolla, 2016 (40)	5	Dep	45.2 ± 14.4	TSM	D	6
Riva-Posse, 2014 (41)	14	Dep	42.0 ± 8.9	TSM	P	24
Lujan, 2013 (42)	1	Dep	n.r.	TSM	P	12
Lujan, 2012 (43)	7	Dep	42.4 ± 13.3	TSM	D	15–41
Coenen, 2015 (44)	1	Pain	n.r.	DTT	D	15
Hunsche, 2013 (45)	4	Pain	60.8 ± 13.6	DTT	D	12
Boccard, 2016b (46)	8	Pain	53.4 ± 6.0	TSM	P	n.r.
Kim, 2016 (47)	5	Pain	56.0 ± 14.8	TSM	P	2–48
Kovanliyaka, 2014 (48)	1	Pain	44	TSM	D	12
Owen, 2007 (49) and Owen, 2008 (50)	4	Pain	n.r.	TSM	P	n.r.

*n.r., not reported*.

*Indications. Tre, tremor; PD, Parkinson’s disease; ET, essential tremor; Dys, dystonia; HI, head injury; FOG, freezing of gait; OCD, obsessive–compulsive disorder; Dep, depression*.

*Approaches. DTT, direct tract targeting; TPA, tract proximity analysis; TSM, tract stimulation modeling*.

*Methods. D, deterministic; P, probabilistic*.

*^a^Ages are expressed as mean ± SD*.

*^b^Two underwent DBS while four had thalatomy*.

*^c^Excluded two patients who also appeared in the study by Coenen et al. (20) and Coenen et al. (21)*.

*^d^Excluded two patients who did not have tremor symptoms preoperatively*.

### Movement Disorders

A total of 80 patients with movement disorders were treated using DBS combined with a DTI-based approach leading to an overall mean percentage improvement in symptoms of 68.7% (SD = 16.0%). The main indication for DBS was medically refractory tremor (14 studies, *n* = 69) mainly due to either Parkinson’s disease or essential tremor, followed by gait dysfunction (one study, *n* = 1) and dystonia (two studies, *n* = 10).

#### Medically Refractory Tremor

Deep brain stimulation achieved an overall mean improvement of 70.0% (SD = 16.5%) over a follow-up of 11.8 months (SD = 6.5) in 14 studies with a total of 69 patients (mean age 62.5 ± 7.4 years) with treatment refractory tremor (Table 2).

**Table 2:** Deep brain stimulation with diffusion tensor imaging for tremor.

First author (ref)	Response	Scale	Mean preop, score (range)	Mean postop, score (range)	Mean improvement (%)	Target
**Prospective direct tract targeting**						

Fenoy (16)	20 responders	TETRAS (head)	2.3 ± 0.5 (2–3)	0.8 ± 0.4 (0–1)	63.9 ± 19.5	VIM
		TETRAS (right arm)	2.6 ± 0.5 (2–3.5)	0.8 ± 0.5 (0–1.5)	71.7 ± 18.4	
		TETRAS (left arm)	2.7 ± 0.5 (2–4)	0.8 ± 0.6 (0–1.5)	71.9 ± 20.6	
Coenen (17)	1 responder	ETRS	51	20	61	VIM
O’Halloran (18)	2 responders	UPDRS–III global	41.0 ± 46.7 (8–74)	8.5 ± 5.0 (5–12)	60.6 ± 32.5	cZI^a^
Coenen (19)	2 responders	UPDRS–III global	59.5 ± 5.0 (56–63)	Both 11	81.4 ± 1.5	STN
		UPDRS–III tremor	13.5 ± 3.5 (11–16)	2.5 ± 0.7 (2–3)	81.5 ± 0.4	
Coenen (20)	1 responder	ETRS	63	22	65	VIM
Coenen (21)	1 responder^b^	ETRS	11	9	18	VIM
		Head tremor			90	

**Retrospective tract proximity analyses**						

King (22)/Sammartino (23)	6 responders	CRST B	19.2 ± 5.7 (11–28) 17.8 ± 5.4	7.8 ± 2.6 (5–11)	56.9 ± 15.5 56.0 ± 11.9 (overall) 78.0 ± 17.2 (op side)	VIM
		TETRAS				
Coenen (24)	8 responders	CRST	49.6 ± 12.8 (36–68)	12.4 ± 7.4 (3–23)	76.7 ± 10.9	VIM
	1^c^ non-responder		35	21	40.0	
Sweet (25)	4^d^ responders 3 non-responders	UPDRS–III tremor	8.2 ± 3.1 (5.5–11.5) 7.5 ± 4.2 (3–13)	6.3 ± 2.1 (4–8) 1.1 ± 1.4 (0–3)	87.8 ± 17.4 21.3 ± 12.5	STN
Coenen (26)	1 responder	ETRS UPDRS–III global UPDRS–III tremor	33 27 7	9 17 1	72.7 37.0 (stopped meds) 85.7 (stopped meds)	VIM

**Retrospective tract stimulation modeling**						

Boccard (27)	1 responder	CRST	25	7	72.0	VOP-ZI
Klein (28)	12 responder	FTM A + B	46.2 ± 16.5 (18–77)	13.6 ± 8.6 (3–33)	71.8 ± 11.8	VIM
Pouratian (29)	6 responder	FTM global FTM A FTM B	53.7 ± 24.0 (9–73) 10.0 ± 6.3 (2–19) 5.4 ± 2.7 (2–10)	32.8 ± 24.0 (9–66) 5.9 ± 4.5 (0–15) 2.1 ± 1.4 (0–4)	39.0 41.0 61.0	VIM

Prospective cohort:			Retrospective cohort:			
Overall responder proportion = 27 out of 27 (100%)			Overall responder proportion = 38 out of 42 (90%)			
Overall mean improvement in responders = 71.7 ± 17.9%			Overall mean improvement in responders = 73.1 ± 11.6%			
Overall mean improvement among non-responders = nil			Overall mean improvement among non-responders = 31.0 ± 13.2%			

*Scales. TETRAS, The Essential Tremor Rating Assessment Scale (51); ETRS, Essential Tremor Rating Scale; UPDRS-III, Unified Parkinson’s Disease Rating Scale part III measures motor signs of PD (52); CRST, clinical rating scale for tremor (53); FTM, Fahn-Tolosa-Marin tremor rating scale (54); FTM A measures tremor severity; FTM B measures task-related tremor*.

*Targets. cZI, caudal zona incerta; STN, subthalamic nucleus; VIM, ventral intermediate thalamus; VOP-ZI, ventral oral posterior thalamic nucleus-zona incerta*.

*^a^One patient had initial right-sided lead implanted within cZI, but the DRT (with an atypical anterior trajectory) was outside of field of stimulation. Patient experienced stimulation-induced dystonic symptoms and the lead was replaced with one implanted in a more anterior position within DRT, giving good tremor relief with reduced side effects*.

*^b^Patient demonstrated excellent head tremor control (>90%) which was not well expressed by 18% improvement in ETRS*.

*^c^Patient showed 42% improvement in ETRS but had complicated ET with “yes-yes”-type of head tremor which was hard to judge intraoperatively*.

*^d^Excluded two patients who did not have tremor symptoms preoperatively*.

In six prospective studies, 27 patients underwent DBS of the ventral intermediate thalamic nucleus (VIM) (16–21), subthalamic nuclei (STN) (19), or caudal zona incerta (cZI) (18) (Table 2) where the dentatorubrothalamic (DRT) tract was simultaneously targeted using DTI and verified with imaging analyses postoperatively. All patients had good response (100% responders) with a mean improvement of 71.7% (SD = 17.9%) over a follow-up period of 13.6 months (SD = 9.5). One patient who achieved 37.5% moderate improvement in the UPDRS-III demonstrated more than 90% reduction in baseline tremor by 6 months postoperatively (19). Another patient experienced excellent head tremor control (>90%) which was not well represented by 18% improvement based on ETRS scores (21).

In eight studies (22–30) where DTI was used in *post hoc* analysis, the overall mean improvement with DBS was 68.5% (SD = 15.7%) over a mean follow-up period of 8.8 months (SD = 3.1), with 38 out of 42 patients (90%) achieving good response (Table 2). The overall mean improvement between responders and non-responders were 73.1% (SD = 11.6%) and 31.0% (SD = 13.2%), respectively. The responder rate was therefore higher in the prospective DTI group (100%) compared to the retrospective DTI group (90%).

Diffusion tensor imaging was used in the *post hoc* analysis of the DRT tract proximity to the implanted electrode contacts in five studies with 25 patients who underwent stimulation of the VIM or STN (22–26). Analysis of electrode proximity to the DRT tract found that effective electrodes were localized in close proximity of or within the DRT tract itself among the good responders, with an optimal position of the active contact located just anterior to or at the center of the DRT (24). There was a non-significant trend toward improved efficacy with increasing proximity of the DRT tract and the electrode contact or stimulated electrical fields (24, 25). Ineffective contacts tended to be located outside the anterior border of the DRT tract and further from the center (3 vs. 1.97 mm) (24). Postoperative tremor control tended to be better when the DRT was closer to the volume of tissue activated by the effective contact (25).

Differences in connectivity patterns of effective and non-effective electrode contacts to the cortical and subcortical areas were analyzed among 19 patients in another four studies (27–29). Effective contacts for tremor control were found to have stronger structural connectivity to the superior frontal gyrus in Parkinson’s disease patients (27) and primary motor cortex in essential tremor patients (28, 29). Klein et al. (28) observed in 12 patients with tremor that there was a smaller volume of precentral gyrus connectivity with ineffective electrodes compared to effective electrodes, and consequently a weaker structural connectivity to the premotor cortex.

#### Gait Dysfunction in Parkinson’s Disease

In a single patient study for Parkinson’s disease-related on-state freezing of gait, DBS was performed with direct targeting of the pedunculopontine nucleus (PPN) with the aid of DTI FA mapping (30). The patient demonstrated 42% improvement in a freezing of gait questionnaire score and 14% improvement in the gait and falls questionnaire postoperatively. Comparison of pre- and post-DBS imaging revealed a normalization effect on pathological PPN connectivity with DBS. In particular, loss of pre-DBS cerebellar connectivity was restored, while overactivity of the corticopontine fibers in the anterior pons was reduced postoperatively. Additionally, dominant connectivity with motor cortex observed pre-DBS was reduced and connectivity with other prefrontal areas such as the primary motor cortex became dominant postoperatively. The authors suggest that DBS may affect reorganization in the topography of connectivity and neuroplasticity.

#### Medically Refractory Dystonia

Deep brain stimulation of the globus pallidus internus (GPi) in 10 patients (mean age 59.1 ± 14 years) with medically intractable dystonia achieved excellent response in all patients, with an overall mean improvement of 79.5% (SD = 12.0%) on the Burke–Fahn–Marsden Dystonia Rating Scale, part II subscale (31, 32) over a follow-up period between 2 and 4 months. Retrospective DTI analysis (31) revealed that clinically effective ventral GPi electrodes had stronger connectivity to posterior cortical areas in the primary sensory cortex and posterior motor cortical regions, while dorsal GPi (less clinically efficient) were more connected to anterior cortex in motor and premotor regions. Rozanski et al. (32) extended their work in the same group of patients by showing a close anatomic vicinity of the clinically efficient DBS electrodes and the pallidothalamic tract, in particular the *ansa lenticularis*, providing support for the pallidothalamic tracts as DBS target for dystonia.

### Medically Refractory OCD

In three studies with nine patients (mean age 36.7 ± 9.0 years) with treatment refractory OCD, DBS achieved an overall mean reduction of 36.5% (SD = 19.1%) on the Yale-Brown Obsessive–Compulsive Scale (Y-BOCS) over a mean follow-up period of 19.3 months (SD = 7.2) (33–35) (Table 3).

**Table 3:** Deep brain stimulation with diffusion tensor imaging for obsessive–compulsive disorder.

First author (ref)	Response	Scale	Mean preop, scores (range)	Mean postop, scores (range)	Mean improvement (%)	Target	Tract
**Prospective direct tract targeting**							

Coenen (33)	2 responders	Y-BOCS	34.5 ± 6.4 (30–39)	20.5 ± 7.8 (15–26)	41.7 ± 11.79	VC/VS	slMFB

**Retrospective tract proximity analysis**							

Makris (34)	1 responder	Y-BOCS	Not reported	Not reported	35	VC/VS	lOFC-thal mOFC-thal

**Retrospective tract stimulation modeling**							

Hartmann (35)	4 responders 2 non-responders	Y-BOCS	Not reported	Not reported	53.8 ± 27.9 15.8 ± 17.5	ALIC NAcc	–

Prospective cohort:				Retrospective cohort:			
Overall responder proportion = 2 out of 2 (100%)				Overall responder proportion = 5 out of 7 (71%)			
Overall mean improvement in responders = 41.7 ± 11.8%				Overall mean improvement in responders = 44.4 ± 27.9%			
Overall mean improvement among non-responders = n.a.				Overall mean improvement among non-responders = 15.8 ± 17.5%			

*Scale. Y-BOCS, Yale-Brown Obsessive–Compulsive Scale (55)*.

*Targets. VC/VS, ventral anterior internal capsule and ventral striatum; ALIC, anterior limb of internal capsule; NAcc, nucleus accumbens*.

*Tract. l/mOFC-thal, lateral/medial orbitofrontocortical-thalamic*.

Deep brain stimulation of the anterior limb of the internal capsule (ventral capsule) and ventral striatum (VC/VS) was performed in two patients with simultaneous targeting of the superolateral branch of the medial forebrain bundle (slMFB) with the aid of DTI (33). Both patients showed good response with a mean improvement of 41.7% (SD = 11.8%) at 12 months.

In two studies (34, 35) where DTI was used in *post hoc* analysis, the overall mean improvement with DBS was 34.8% (SD = 22.7%) over a mean follow-up period of 21.4 months (SD = 6.8), with five out of seven patients (71%) achieving good response. The overall mean improvement among responders and non-responders were 44.4% (SD = 27.9%) and 15.8% (SD = 17.5%), respectively.

Retrospective tract proximity analysis was performed for a patient who underwent DBS of the VC/VS with 34.8% reduction in Y-BOCS (34). The study found that ineffective contacts only engage either one or none of the lateral or medial orbitofrontal cortex-thalamic connecting tracts. The authors proposed a tractography-guided patient-specific approach in DBS targeting the “center of mass” of orbitofrontal cortex-thalamic connections.

Computational models were used to stimulate the activation of fiber tracts by the DBS electrodes in six patients who underwent DBS of the anterior limb of the internal capsule and nucleus accumbens (35). Large fiber activation were observed in the right middle (anterior part) frontal gyrus in patients with best response (mean improvement 77%), superior frontal gyrus in the moderate responders (mean improvement 30.5%) and right thalamus and orbital part of the inferior frontal gyrus in the non-responders (mean improvement ≤ 5%).

### Medically Refractory Depression

Deep brain stimulation for treatment refractory depression achieved an overall mean improvement of 48.3% (SD = 40.0%) over follow-up period of 21.1 months (SD = 14.9) in eight studies with a total of 40 patients (mean age 46.4 ± 11.1 years) (36–43) (Table 4).

**Table 4:** Deep brain stimulation with diffusion tensor imaging for depression.

First author (ref)	Response	Scale	Preop scores	Postop scores	Mean improvement (%)	Target
**Prospective direct tract targeting**						

Fenoy (36)	2^a^ responders 1 non-responder	MADRS HDRS29 MADRS HDRS29	32.5 ± 3.5 (30–35) 41.5 ± 0.7 (41–42) 34 37	5.0 ± 1.4 (4–6) 11.0 ± 11.3 (3–19) 30 28	84.8 ± 2.7 73.7 ± 26.8 12 24	slMFB
Schlaepfer (37)	6 responders	MADR HDRS24	29.0 ± 8.4 (15–39) 23.0 ± 1.7 (21–25)	7.7 ± 4.3 (2–13) 13.0 ± 7.4 (2–21)	71.4 ± 17.4 44.0 ± 31.7	slMFB
	1 non-responder	MADR HDRS24	35 23	31 25	11 −9	

**Retrospective tract stimulation modeling**						

Tsolaki (38)	1 responder	MADRS	Not reported	Not reported	84	SCC
	1 non-responder		Not reported	Not reported	8	
McNab (39)	1 non-responder	BDI-II	41	Not reported	No effect	SACC
Accolla (40)	1 responder	HAMD24 BDI	28 34	7 12	75.0 64.7	Posterior gyrus rectus
	4^b^ non-responders	HAMD24 BDI	28.8 ± 3.6 (24–32) 42.8 ± 11.1 (30–57)	29.0 ± 5.7 (21–33) 42.3 ± 9.6 (31–52)	−3.4 ± 30.6 −1.2 ± 9.4	SCC
Riva-Posse (41)^c^	12 responders 2^d^ non-responders	HDRS17 BDI-II	23.9 ± 0.7 38.4 ± 2.1	(*n* = 7) HDRS < 8 (*n* = 5) HDRS < 15 HDRS > 15	>50 <50	SCC
Lujan (42)	1 responder	HDRS17	23	5	78.3	SCC
Lujan (43)	5 responders	HDRS MADRS	32.0 ± 3.5 (26–35) 30.6 ± 5.3 (25–37)	2.6 ± 3.6 (0–9) 0.6 ± 1.3 (0–3)	92.0 ± 10.7 98.4 ± 3.6	VC/VS
	2 non-responders	HDRS MADRS	32.0 ± 7.1 (27–37) 30.0 ± 2.8 (28–32)	30.5 ± 4.9 (27–34) 21.5 ± 4.9 (18–25)	4.1 ± 5.7 28.8 ± 9.8	

Prospective cohort:				Overall responder proportion = 8 out of 10 (80%)		
Overall mean improvement in responders = 68.0 ± 10.0%				Overall mean improvement among non-responders = 11.6 ± 0.2%		
Retrospective cohort:				Overall responder proportion = 20 out of 30 (67%)		
Overall mean improvement in responders = 83.9 ± 3.6%				Overall mean improvement among non-responders = 8.3 ± 15.4%		

*Scale. MADRS, Montgomery-Asberg Depression Rating Scale (56); HDRS29/HDRS24/HDRS17/HAMD24, Hamilton Depression Rating Scale (57, 58); BDI/BDI-II, Beck Depression Inventory (59, 60)*.

*Targets. SCC, subcallosal cingulate cortex; VC/VS, ventral capsule/ventral striatum; SACC, subgenual anterior cingulate cortex*.

*^a^Excluded one patient who withdrew before follow-up at 26 weeks. MADRS scores at postop 1 week showed 51% improvement*.

*^b^Two requested for removal of DBS system within 12 months*.

*^c^Outcomes were referenced from Holtzheimer et al. (61)*.

*^d^Two patients were explanted before 24 months*.

In prospective studies where the slMFB was directly targeted during DBS with DTI in 10 patients for treatment resistant depression, there was an overall mean improvement in MADRS of 61.6% (SD = 34.9%) (36, 37). The overall improvement among responders (*n* = 8) and non-responders (*n* = 2) were 68.0% (SD = 10.0%) and 11.6% (SD = 0.2%), respectively. DTI analyses revealed that the final electrode locations were found to be in close proximity or within the slMFB among the eight responders. In one non-responder, the final electrode was localized in the slMFB but weaker connectivity was found between the MFB and the prefrontal cortex compared to the responders (36). Analysis results of the electrode location or connectivity was not reported in the remaining non-responder (37).

In six studies (38–43) where DTI was used in *post hoc* analysis, the overall mean improvement with DBS was 43.0% (SD = 44.0%) over a mean follow-up period of 8.8 months (SD = 3.1), with 20 out of 30 patients (66.7%) achieving good response (Table 4). The overall mean improvement among responders and non-responders were 83.9% (SD = 3.6%) and 8.3% (SD = 15.4%), respectively.

Differences in connectivity patterns of electrode contacts to cortical and subcortical areas were analyzed in 30 patients (37–43). DBS of the subgenual anterior cingulate cortex did not lead to any therapeutic effect in a single patient study (39). DTI analyses in the non-responder revealed that connectivity in amygdalar-thalamic and amygdalar-SACC was found to be disrupted.

In three studies, retrospective analyses of the connectivity of DBS electrode to cortical regions were performed in 19 patients who underwent DBS of the subcallosal cingulate (40–42). Higher connectivity in the medial prefontal areas and ventral caudate was observed while lower connectivity to middle and posterior cingulate were observed in the patient with good response who underwent DBS in the posterior gyrus rectus was compared to four non-responders who had DBS in the subcallosal cingulate (40). Common pathways connecting the medial frontal cortex, cingulate cortex and subcortical nuclei was seen in responders, but not in non-responders (41). In a good responder, the number of axons within the same pathway activated by the effective electrode, which produced the best therapeutic effects, was significantly higher than the sub-therapeutic electrode contacts (42). Tsolaki et al. (38) used tractography to identify the optimal site of stimulation within the subcallosal cingulate cortex, and further demonstrated that compared to non-responder, the DBS electrodes in the responder were implanted and stimulation delivered closer to the tractography-optimized target.

### Medically Refractory Pain Disorders

A total of 23 patients (mean age 55.2 ± 10.7 years) in seven studies underwent DBS for chronic pain (44–50) (Table 5) with an overall improvement of 49.7% (SD = 35.1%).

**Table 5:** Deep brain stimulation with diffusion tensor imaging for pain disorders.

First author (ref)	Response	Scale	Preop scores	Postop scores	Mean improvement (%)	Target	Tract
**Prospective direct tract targeting**							

Coenen (44)	1 responder	VAS	7–9	2–5	44–78	PVG, PAG, VCP	MPNS/ATR IC/STP
							TL/ML
Hunsche (45)	3 responders	VAS	All 10	4.7 ± 2.3 (2–6)	53.3 ± 23.1	PLIC	STC
	1 non-responder			9	10		

**Retrospective tract stimulation modeling**							

Boccard (46)	6 responders	VAS	8.4 ± 1.4 (6–10)	Not reported	Analgesic relief in all, with 1 pain free	ACC	–
	2^a^ non-responders		9–10	Not reported	–		
Kim (47)	4 responders	VAS	Not reported	Not reported	Moderate to significant	PAG, VPL, VPM	–
	1 non-responder				No relief		
Kovanliyaka (48)	1 responder	–	–	Pain free	100	VPL	–
Owen (49, 50)	2 responders	MPQ	20.0 ± 12.7 (11–29)	Both 0	100	PVG, PAG	–
	2^b^ non-responders		41.5 ± 19.1 (55–28)	43, explanted	22		

Prospective cohort:				Retrospective cohort:			
Overall responder proportion = 4 out of 5 (80%)				Overall responder proportion = 13 out of 18 (72%)			
Overall mean improvement in responders = 51.7 ± 23.1%				(Insufficient quantitative data to calculate overall mean improvement)			
Overall mean improvement among non-responders = 25.0%							

*Scales. VAS, visual analog scale; MPQ, McGill Pain Questionnaire (62)*.

*Targets. PVG, periventricular gray; PAG, periaqueductal gray; VCP, nucleus ventralis caudalis posterior; PLIC, posterior limp of internal capsule; ACC, anterior cingulate cortex; VPL, ventroposterolateral nucleus; VPM, ventroposteromedial nucleus*.

*Tracts. MPNS/ATR, median polysynaptic pain system/anterior thalamic radiation; IC/STP, internal capsule/superior thalamic peduncle; TL/ML, trigeminal lemniscus/medial meniscus; STC, spinothalamocortical tract*.

*^a^One patient requested for DBS system removed and one did not have the internal pulse generator implanted*.

*^b^System was explanted in one patient (non-responder) after trial due to poor efficacy*.

Diffusion tensor imaging was used prospectively to target white matter tracts of interest in five patients (44, 45) a mean improvement was 42.8% (SD = 23.1%) with four out of five (80%) achieving good response. The mean improvement in responders and non-responder are 51.7 ± 23.1 and 25%, respectively.

In a single patient study where DBS to periventricular/periaqueductal gray (PVG/PAG) was performed, DTI was used to target the median polysynaptic pain system and trigeminal lemniscus/medial lemniscus (44). The final electrode position was verified postoperatively and the patient had good pain relief postoperatively. In another four patients, DTI was used for targeting of the spinothalamocortical tract in addition to the posterior limb of internal capsule (45). Only three reported good pain relief while stimulation failed to reach any long-lasting positive effects for one patient. Postoperative imaging showed the electrodes localized in the intended locations in all patients and reconstruction of the spinothalamocortical tract did not reveal any differences between the non-responder and other responders in terms of electrode location (45).

Diffusion tensor imaging was used in the retrospective analyses of the electrode connectivity to remote brain regions (46–50), with 13 out of 18 patients (72%) achieving good response. In a group of eight patients where the anterior cingulate cortex (ACC) was stimulated, DTI analyses showed responders (*n* = 6) had stronger connectivity to the anterior thalamus and brain stem (through the medial forebrain bundle/anterior thalamic radiation), insula, and superior middle frontal gyrus while non-responders (*n* = 2) had stronger connectivity to the precuneus and cingulum (46).

Periventricular gray/PAG was targeted in eight patients (six responders), among whom four also received simultaneous stimulation in the ventroposterolateral and ventroposteromedial nuclei (VPL/VPM) (47, 49, 50). Those with suboptimal outcomes (one non-responder and one responder who subsequently failed) had electrodes which were found to be too medial to the VPL/VPM region (47).

### DTI Use in Revision Surgery

Further evidence for tractography-based DBS surgery have been found in cases where revision or removal of an initially implanted leads using conventional methods led to the electrode location or stimulation being outside the DRT. Revision of electrodes based on direct tract targeting of the DRT tract resulted in improved efficacy of more than 50%. For instance, O’Halloran et al. (18) reported a 61-year-old patient who underwent bilateral DBS of cZI. He experienced good initial response (>90%) but subsequently developed dystonia of left leg and speech slurring. Tract proximity analyses showed a right-sided lead slightly posterior to DRT tract (but still well within cZI). The right DRT tract had an atypical anterior trajectory and was located outside the field of stimulation of the DRT tract. The lead was revised to a more anterior location within the field of stimulation and closer to the STN, leading to 84% improvement in tremor control without complication.

Coenen et al. (19) reported a 75-year-old patient with equivalent-type idiopathic Parkinson’s syndrome who underwent bilateral DBS to STN performed using the conventional approach. He experienced overall symptoms improvement of 54% but was unsatisfied with the extent of right upper extremity tremor reduction. Tract proximity analysis revealed the electrode tip was in close proximity but barely touching the DRT tract. The system was subsequently explanted 2 months later due to infection and a second implantation was performed with a parietal approach. The newly placed electrode localized in and stimulated both DRT tract and STN simultaneously, leading to 62% improvement in UPDRS-III, 78% improvement in tremor, and 70% marked reduction of medication.

In another case report, a 73-year-old woman with essential tremor initially underwent bilateral thalamic DBS of the VIM with conventional targeting methods and intraoperative test stimulation (20). Monolopar stimulation was insufficient for left sided tremor control and a change to bipolar stimulation led to moderate tremor control with stimulation induced gait instability. She underwent a second surgery to have additional right-sided electrode implantation aimed directly at the DRT tract. Combined stimulation of both right sided electrodes led to sufficient 65% tremor control.

Coenen et al. (17) reported a patient with proximal and trunkal “yes-yes”—tremor who underwent conventional bilateral DBS of the VIM and experienced initial beneficial effects. Tremor reoccurred after the microlesioning effect subsided and there was no marked improvement with adjustments over time. The electrodes were explanted. He underwent DTI-assisted DBS revision surgery targeting the DRT in the subthalamic region and showed immediate and sustained improvement of 61% up to 1 year. Analysis of cortical connections revealed both implantations involved the DRT but had different patterns of fiber projections. The initial DBS showed less selective activation of fibers projecting to dorsal prefrontal and supplementary motor region (more anterior and medial). The electrodes in the second implantation revealed more lateral and posterior projections and greater involvement of the precentral and postcentral gyrus.

A patient who presented with neuropathic trigeminal pain syndrome after repeated resection of an epidermoid tumor involving the trigeminal ganglion was reported by Coenen et al. (44). The patient had DBS to the PV/PAG and sensory thalamus which did not provide pain relief clinically (VAS 7–9). The previously implanted electrodes were seen touching the median polysynaptic pain system on MR imaging and subsequently explanted. New DBS electrodes were implanted in the PV/PAG and VCP, and diffusion tractography revealed they stimulated both the lateral and medial (internal capsule/superior thalamic peduncle and trigeminal and medial lemniscus) systems, leading to good outcome (VAS 2–5) over 15 months.

## Discussion

Although majority of the studies of earlier studies were retrospective in nature and have provided a strong basis for the utility of integrating diffusion tractography into DBS planning. More recent studies have used DTI prospectively and have tended to show a greater response rate compared to the retrospective studies. These reported improved response rates have been more apparent in studies of essential tremor and depression.

In the retrospective studies, patients in whom the DBS electrodes were within the DTI targets based on tract proximity analysis experienced better outcomes than those in whom the electrodes were not. Case series of unsuccessful direct tract targeting in essential tremor and Parkinson’s disease patients, for instance, have shown electrodes implanted using the conventional approach and found outside of the DRT tract had unsatisfactory efficacy. Lead revision of these electrodes or additional electrode implantation targeted at DRT tract prospectively consistently led to significantly improved tremor control (17–20). In such cases which are in effect direct comparison between conventional versus DTI-based targeting, the DTI-based approach appeared to provide better outcome.

In other cases, the electrode was localized in the putative correct position but stimulation did not lead to expected efficacy. Tract stimulation modeling revealed that there were significant differences in connectivity to specific cortical and subcortical areas (36). Beyond accurate targeting of the structures and white matter tracts, DTI can be used to evaluate white matter connectivity between putative targets and subcortical or cortical areas as these appear to have a significant importance in determining good outcome. Analysis of this structural connectivity could inform preoperative decision making and potentially predict treatment outcomes.

Our review show that the most commonly used method in prospective studies for surgical planning was based on the deterministic algorithm. The use of probabilistic tractography was mainly used for tract proximity or simulation in retrospective studies. In the article by Sammartino et al. (23), a comparison was performed comparing deterministic against probabilistic tractography for the DRT in six patients with essential tremor. The authors report equivalent results using either probabilistic or deterministic techniques. The higher structural connectivity obtained through probabilistic tracking did not translate into more accurate target definition of the VIM over the deterministic method. Therefore, for certain well-known tracts such as the slMFB or DRT, it would appear that the deterministic tractography using currently commercially available software would be sufficient for target definition without having to use the more computationally and time intensive probabilistic method.

Many of the current studies are limited by their small sample size or retrospective nature. However, it would appear that DTI has utility in patient-specific anatomical targeting that can be used to increase the efficacy of DBS surgery. Furthermore, connectivity studies can also be used to predict outcome from DBS surgery. More recent prospective studies are also showing increased response rates compared to the initial retrospective studies. The use of DTI in DBS planning appears to be underutilized and further studies are warranted given that surgical outcome can be optimized using this non-invasive technique.

## Author Contributions

All authors have made a substantial, direct, and intellectual contribution to the work and approved it for publication.

## Conflict of Interest Statement

The authors declare that the research was conducted in the absence of any commercial or financial relationships that could be construed as a potential conflict of interest.
